# Direct Targeting of the Raf-MEK-ERK Signaling Cascade Inhibits Neuroblastoma Growth

**DOI:** 10.3390/curroncol29090512

**Published:** 2022-09-10

**Authors:** Rameswari Chilamakuri, Saurabh Agarwal

**Affiliations:** Department of Pharmaceutical Sciences, College of Pharmacy and Health Sciences, St. John’s University, New York, NY 11439, USA

**Keywords:** pediatric cancer, neuroblastoma, CI-1040, PD184352, signaling pathway, drug repurposing

## Abstract

The Raf-MEK-ERK signaling network has been the subject of intense research due to its role in the development of human cancers, including pediatric neuroblastoma (NB). MEK and ERK are the central components of this signaling pathway and are attractive targets for cancer therapy. Approximately 3–5% of the primary NB samples and about 80% of relapsed samples contain mutations in the Raf-MEK-ERK pathway. In the present study, we analyzed the NB patient datasets and revealed that high *RAF* and *MEK* expression leads to poor overall survival and directly correlates with cancer progression and relapse. Further, we repurposed a specific small-molecule MEK inhibitor CI-1040 to inhibit the Raf-MEK-ERK pathway in NB. Our results show that CI-1040 potently inhibits NB cell proliferation and clonogenic growth in a dose-dependent manner. Inhibition of the Raf-MEK-ERK pathway by CI-1040 significantly enhances apoptosis, blocks cell cycle progression at the S phase, inhibits expression of the cell cycle-related genes, and significantly inhibits phosphorylation and activation of the ERK1/2 protein. Furthermore, CI-1040 significantly inhibits tumor growth in different NB 3D spheroidal tumor models in a dose-dependent manner and by directly inhibiting spheroidal tumor cells. Overall, our findings highlight that direct inhibition of the Raf-MEK-ERK pathway is a novel therapeutic approach for NB, and further developing repurposing strategies using CI-1040 is a clinically tractable strategy for effectively treating NB.

## 1. Introduction

Neuroblastoma (NB) is the second most common pediatric solid tumor and is derived from the peripheral sympathetic nervous system. NB mostly affects children below 5 years of age and, in some rare cases, young adolescents and adults. NB is clinically and biologically heterogeneous due to alterations in signaling regulators such as *MEK* and transcription factors amplification such as *MYCN* [1]. Current therapeutic approaches include combinational chemotherapy, surgery, radiation, retinoid therapy, immunotherapy, tyrosine kinase inhibitors, and angiogenesis inhibitors [2,3,4,5]. Current therapies have overall poor outcomes and cause acute life-threatening toxicities and chronic illnesses. Therefore, identifying molecular mechanisms involved in NB pathogenesis and progression and developing novel therapeutic approaches targeting these mechanisms is important to effectively treating NB [6].

The Mitogen-activated protein kinase (MAPK) pathway is one of the most important cell signaling pathways known to dysregulate in different cancers, including breast, thyroid, non-small cell lung cancer, and NB [6,7,8,9]. Approximately 3–5% of the primary NB samples and about 80% of relapsed samples contain mutations in the Ras-Raf-MEK-ERK pathway [10]. Activation of the MAPK pathway is initiated by the extracellular signal binding to the receptor tyrosine kinase and activating the Ras proteins (NRAS, HRAS, and KRAS). Activation of Ras to Ras-GTP is tightly controlled by the activation of both growth factor receptor-bound protein 2 (Grb2) and son of sevenless (SOS) proteins and by forming a Grb2-SOS complex [11]. Ras-GTP binds to and activates Raf, which phosphorylates and activates MEK protein [12]. Activated MEK phosphorylates and activates ERK that further dimerizes and translocates to the nucleus to promote the activity of various transcription factors such as CREB, MYC, JUN, FOS, ELK-1, ETS, MSK, and ATF2. These transcription factors are involved in regulating various cellular physiological processes such as cell proliferation, metabolism, differentiation, cell cycle progression, and metabolism [13,14]. Activated ERK is also known to further activate the upstream regulators, such as MEK and Raf, through the negative feedback mechanism [15]. Mutations and oncogenic activation of the MAPK pathway proteins are well known in different cancers, including NB [1,16,17,18,19]. MEK expression is high in NB cell lines. Previous research reported that inhibition of MEK activation to inhibit the Ras-MEK-ERK oncogenic pathway inhibited the NB cell proliferation [20,21]. Therefore, targeting the MEK activation to inhibit the Ras-MEK-ERK oncogenic pathway is an effective therapeutic approach for targeted cancer therapy.

To date, four MEK inhibitors trametinib, binimetinib, selumetinib, and cobimetinib received FDA approval for treating different cancers, including melanoma and neurofibromatosis type I tumors [7]. In addition, several MEK/ERK inhibitors such as BVD-523, CC-90003, FCN-159, refametinib, SHR7390, E6201, CC-90003, KO-947, TAK-733, AZD8330, and LY3214996 are currently in clinical trials for treating pancreatic adenocarcinoma, breast cancer, BRAF-mutant colorectal cancer, hepatocellular cancer, metastatic melanoma, non-small-cell lung cancer, and NB [22]. In addition, trametinib (NCT03434262 and NCT02124772) and selumetinib (NCT03213691) are currently in phase 1 and phase 2 clinical trials to treat NB [23]. CI-1040 is an orally active small-molecule inhibitor developed by Pfizer Inc. that binds to a hydrophobic pocket of MEK and locks it into a closed catalytically inactive form. Due to the inactivation of MEK, CI-1040 also inhibits the phosphorylation of ERK1/2 and overall inhibits the MAPK pathway [24,25,26,27]. CI-1040 has been shown to inhibit different cancers, including pancreatic, colon, breast, and non-small-cell lung carcinoma [27,28,29]. In addition, CI-1040 is the first MEK-targeted agent to enter clinical trials and has been reported as a well-tolerated drug in phase 1 and 2 clinical trials (NCT00034827) [28,29].

In the present study, we repurposed the CI-1040 in NB and demonstrated that disruption of the Raf-MEK-ERK pathway has a profound effect on inhibiting NB growth. Our results show that CI-1040 inhibits NB proliferation in 2D cell culture and 3D spheroid tumor models by inducing apoptosis and blocking the cell cycle S phase in a dose-dependent manner. Together, our results demonstrate the efficacy of CI-1040 in NB and point toward a novel therapeutic approach by incorporating MEK inhibitors in NB treatment approaches.

## 2. Materials and Methods

### 2.1. Cell Culture and Reagents

Normal fibroblast control cell lines (WI-38, NIH-3T3, and COS-7) and human NB cell lines, MYCN-amplified (NGP, LAN-5, CHLA-255-MYCN), and MYCN-non-amplified (SH-SY5Y, SK-N-AS, CHLA-255), were routinely cultured and maintained as described previously [30]. All the cell lines were tested monthly for mycoplasma and validated via short tandem repeat analysis for genotyping within the past six months. Primary (anti-ERK1/2 (4695S), anti-pERK1/2 (Thr202/Tyr204) (4370S), anti-Cyclophilin B (43603S)), and secondary antibodies (anti-rabbit IgG HRP-linked (7074S)) were acquired from Cell Signaling Technology, Danvers, MA, USA. CI-1040 (PD184352) was acquired from MedChem Express, Monmouth Junction, NJ, USA. CI-1040 is dissolved in DMSO. Control groups in this study were treated with DMSO and used as vehicle control.

### 2.2. Clinical Patient Dataset

Different NB patient datasets were evaluated by using the publicly available R2: Genomic Analysis and Visualization Platform database (https://hgserver1.amc.nl/cgi-bin/r2/main.cgi (accessed on 16 July 2022)). This database supports the multi-parametric analysis of NB patient outcomes and includes microarray profiles of NB patient samples with gene expression data. This database provides Kaplan–Meier long-rank analysis by comparing gene expression to the overall survival of NB patients.

### 2.3. Cell Viability and Clonogenic Assay

CellTiter 96 AQueous One Solution Cell Proliferation Assay (G3582; Promega Corp., Madison, WI, USA) was used to perform cell viability assays according to the manufacturer’s instructions and as described previously [30,31]. NB cells were treated with increasing concentrations of CI-1040 for 72 h, and cell viability was measured at 490 nm using a spectrophotometer (SpectraMax iD3, Molecular Device, San Jose, CA, USA). DMSO was used as vehicle control. GraphPad software (Prism 9, San Diego, CA, USA) is used for data analysis and for calculating IC_50_ values. Clonogenic assays were performed as described previously [31]. Crystal violet solution is used to stain and visualize the colonies. Colony counting software (OpenCFU ver. 3.8) is used to quantify the number of colonies. Control treatment groups were used to normalize and plot the data. Cell proliferation and clonogenic assays were performed at least three times with three technical replicates.

### 2.4. Apoptosis and Cell Cycle Assay

Apoptosis assays were performed using eBioscience Annexin V-FITC Apoptosis Detection Kit (Cat. BMS500FI-300, ThermoFisher Scientific, Waltham, MA, USA), and cell cycle assays were performed using FxCycle PI/RNase Staining Solution (C10633; ThermoFisher Scientific, Waltham, MA, USA) and Click-iT Plus EdU Alexa Fluor 488 Flow Cytometry Assay Kit (ThermoFisher Scientific Cat. C10633). Briefly, NB cells were seeded and treated with increasing concentrations of CI-1040 for 16 h. Apoptosis and cell cycle assays were performed as described previously and according to the manufacturer’s instructions [31]. Apoptosis was measured by quantifying the percentage of the early apoptotic cells (Q3) that are only positive for Annexin V. The percentage of apoptosis was quantified and extrapolated with control. Cell cycle stages were determined by the horseshoe distribution of cells between different stages based on EdU (5-ethynyl-2′-deoxyuridine) conjugated with Alexa flour 488 and PI (propidium iodide). All flow cytometry assays were performed on the Attune Nxt Flow Cytometer (ThermoFisher Scientific) and were analyzed using the Flow Jo software ver. 10 (BD Biosciences, Ashland, OR, USA).

### 2.5. Spheroidal Tumor Assays

The 3D spheroidal assay was performed using 3D spheroidal 96-well microplates (4515; Corning) as per the manufacturer’s instructions and as described previously [30,31]. Briefly, 2.5 × 10^3^ NB cells per well were seeded and incubated for two days or until the spheroid size reached ~300 μm. Similar size spheroids were randomized and treated with increasing concentrations of CI-1040 for 15 days with regular drug replenishment and spheroidal size measurement on every third day. Spheroidal images were captured using a DMi1 light microscope (Leica Microsystems, Buffalo Grove, IL, USA), and spheroidal size was measured using the Leica software suite tools (LASX, Leica Microsystems, Buffalo Grove, IL, USA). Finally, the Viability/Cytotoxicity Assay Kit for Animal Live & Dead Cells (3002; Biotium Inc., Fremont, CA, USA.) was used to fluorescent label the live and dead cells, and the number of live cells in the spheroids was quantified by CellTiter-Glo 3D Cell Viability Assay (G968; Promega Corp., Madison, WI, USA) solution, according to the manufactures instructions [30,31].

### 2.6. RNA Extraction and Quantitative Real-Time RT-PCR

Gene expression analysis was performed using the RT-qPCR method as described previously [30,31]. Briefly, NB cells were treated with different concentrations of CI-1040 for 6 h, followed by total RNA extraction using RNeasy plus mini kit (74134; Qiagen, Germantown, MD, USA) and cDNA synthesis using the cDNA reverse transcription kit (4368814; ThermoFisher Scientific). Further, RT-qPCR reactions for individual genes were performed using cDNA and SYBR Green dye (4385610; ThermoFisher Scientific). RT-qPCR reactions were performed using QuantStudio 3 Real-Time qPCR System (ThermoFisher Scientific). GAPDH is used as a housekeeping gene to normalize the expression of individual genes. All the Primers used in this study are listed in Table 1.

### 2.7. Immunoblotting Assays

Immunoblotting assays were performed as described previously [30,31]. Briefly, NB cells were treated with increasing concentrations of CI-1040 for 24 h, and the cell pellet was collected and lysed with RIPA buffer (89900; ThermoFisher Scientific) supplemented with phosphatase inhibitor cocktail (PhosSTOP, Roche, Indianapolis, IN, USA) and protease inhibitor cocktail (Complete mini EDTA free, Roche). Protein samples were quantified by Bradford assay (Bio-Rad, Hercules, CA, USA), and 15 μg of protein samples were loaded and separated on a 12% SDS-PAGE gel. Further protein samples were transferred onto the PVDF membrane. ChemiDoc XRS Plus system (Bio-Rad) was used to visualize and document the blots. Densitometric analysis of the protein bands was performed using the ImageJ software ver. 1.8 (Publicly available, NIH).

### 2.8. Statistical Analysis

In the present study, all the biological assays were performed at least three times with three technical replicates. All values are presented as the mean ± standard error (SEM). A two-tailed Student’s *t*-test was used to determine drug treatment groups’ statistical significance after observing the normal distribution. The expression fold difference of individual genes (*p*-values) was calculated by Student’s *t*-test. *p* < 0.05 was considered statistically significant.

## 3. Results

### 3.1. MAP2K2 and RAF1 Expression Strongly Correlate with Poor NB Prognosis

**Figure 1:**
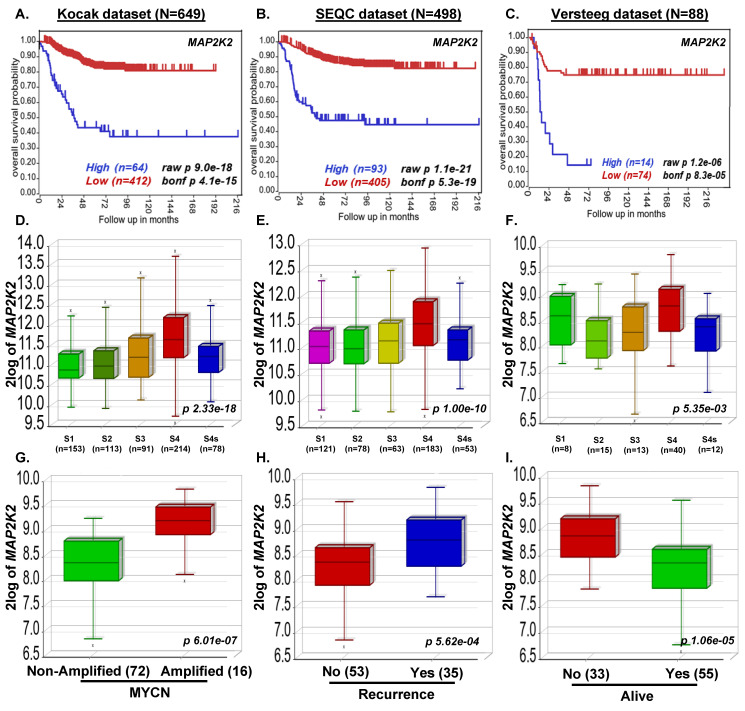
MAP2K2 expression correlates with poor overall survival of NB patients. (**A**–**C**) Kaplan-Meier survival analysis showing high expression of the *MAP2K2* gene leads to the poor overall survival of NB patients in datasets analyzed. (**A**) Kocak dataset (*n* = 649 patients). (**B**) SEQC dataset (*n* = 498 patients). (**C**) Versteeg dataset (*n* = 88 patients). (**D**–**F**) *MAP2K2* expression correlates with NB stage progression in all datasets analyzed. (**D**) Kocak, (**E**) SEQC, (**F**) Versteeg. (**G**–**I**) Versteeg dataset analysis revealed that higher *MAP2K2* expression correlates with (**G**) aggressive MYCN-amplified tumors, (**H**) disease relapse, and (**I**) worst overall outcome.

**Figure 2:**
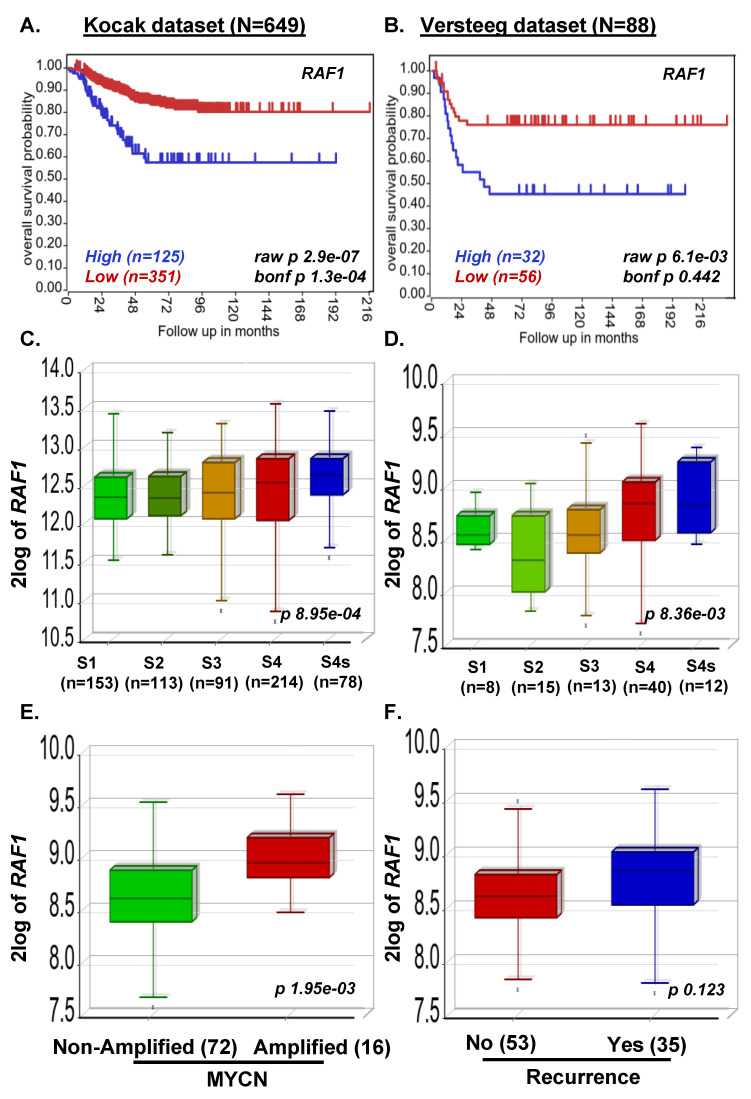
*RAF1* expression correlates with poor overall survival of NB patients. Kaplan-Meier gene expression analysis of *RAF1* shows poor overall survival of NB patients. (**A**) Kocak dataset (*n* = 649 patients). (**B**) Versteeg dataset (*n* = 88 patients). *RAF1* expression correlates with NB stage progression in all patients dataset analyzed. (**C**) Kocak dataset. (**D**) Versteeg dataset. (**E**,**F**) Box-Plot correlation analysis in the Versteeg dataset revealed that higher *RAF1* expression corresponds to (**E**) highly aggressive MYCN-amplified tumors, and (**F**) disease resurrence.

To determine the association of the Ras-MEK-ERK pathway with NB prognosis, we investigated the correlation of *RAF1* and *MAP2K2* gene expression levels with overall NB patient outcomes by analyzing a total of 1235 primary NB patient clinical data. Kaplan–Meier survival analysis revealed that the expression of both *RAF1* and *MAP2K2* is inversely correlated with the overall survival of NB patients. High expression of both *RAF1* and *MAP2K2* showed poor overall survival of NB patients (Kocak *n* = 649, *RAF1 p* = 2.9 × 10^−7^, *MAP2K2 p* = 9.0 × 10^−18^; SEQC *n* = 498, *MAP2K2 p* = 1.1 × 10^−21^; Versteeg *n* = 88, *RAF1 p =* 6.1 × 10^−3^, *MAP2K2 p =* 1.2 × 10^−6^; Figure 1A–C and Figure 2A,B). Further, we observed higher stage NB tumors showed significantly higher *RAF1* and *MAP2K2* expression levels (Kocak *n* = 649, *RAF1 p* = 8.9 × 10^−4^, *MAP2K2 p* = 2.33 × 10^−18^; SEQC *n* = 498, *MAP2K2 p* = 1.00 × 10^−10^; Versteeg *n* = 88, *RAF1 p* = 8.36 × 10^−3^, *MAP2K2 p* = 5.35 × 10^−3^; Figure 1D–F and Figure 2C,D), suggesting that *RAF1* and *MAP2K2* plays a significant role in NB progression. Further, we observed that highly aggressive MYCN-amplified NB tumors have higher expression of *RAF1* and *MAP2K2*, which correlates with disease relapse or reoccurrence conditions in the Versteeg dataset (Figure 1G,H and Figure 2E,F). We further found in Versteeg dataset that high expression of the *MAP2K2* gene leads to the worst outcome of patient death (Figure 1I). These findings suggest that *RAF1* and *MAPK* genes are critical prognostic factors for NB, and higher expression of both *RAF1* and *MAPK* leads to poor survival of NB patients. Therefore, inhibiting the Ras-MEK-ERK pathway is an important therapeutic approach for NB.

### 3.2. CI-1040 Inhibits NB Cell Proliferation and Colony Formation

**Figure 3:**
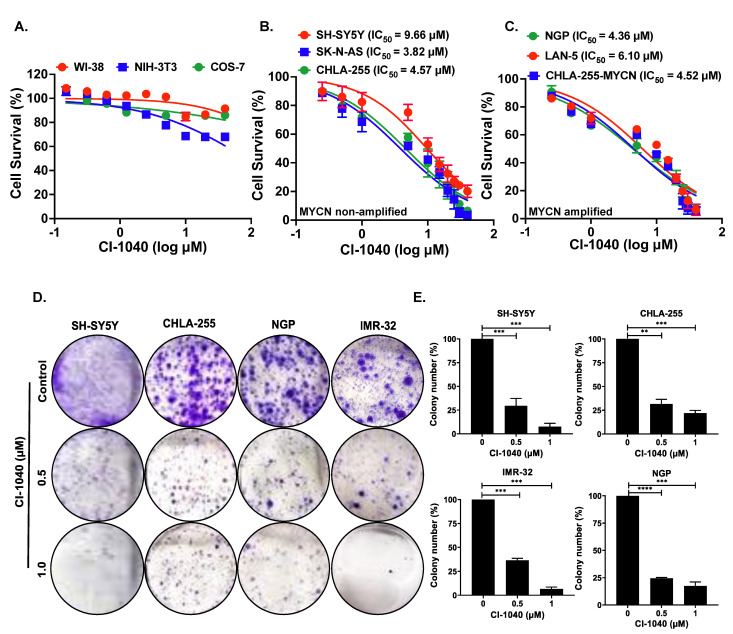
CI-1040 inhibits NB cell proliferation. (**A**–**C**) Cytotoxicity assays were performed in six NB cell lines including 3 MYCN-amplified and 3 MYCN non-amplifies, and 3 non-cancerous fibroblast cell lines, in response to CI-1040. (**A**) Non-cancerous fibroblast cell lines (WI-38, NIH-3T3, COS-7). (**B**) MYCN non-amplified cell lines (SH-SY5Y, SK-N-AS, CHLA-255). (**C**) MYCN amplified cell lines (NGP, LAN-5, CHLA-255-MYCN). IC_50_ values were calculated and shown in parentheses. (**D**,**E**) Colony formation assays were performed in 4 NB cell lines including MYCN-amplified (LAN-5, IMR-32) and MYCN non-amplified (SH-SY5Y, CHLA-255) in response to CI-1040. (**D**) Representative images of colony formation assay in response to CI-1040 treatment in NB cell lines. (**E**) Quantitation of the colony numbers in response to CI-1040 treatment. ** *p* < 0.01, *** *p* < 0.001, and **** *p* < 0.0001.

To inhibit the MAPK pathway, we utilized a specific small-molecule MEK inhibitor, CI-1040. We performed cytotoxicity assays using CI-1040 in six different NB cell lines, including three MYCN-amplified (NGP, LAN-5, CHLA-255-MYCN) and three MYCN non-amplified cell lines (SH-SY-5Y, CHLA-255, SK-N-AS). Additionally, we performed cytotoxicity assays on three normal fibroblast cell lines (WI-38, NIH-3T3, COS-7) to determine the effect of CI-1040 on normal non-cancerous fibroblast cells as controls. Cells were treated with increasing concentrations of CI-1040, and IC_50_ values were determined. Results showed that CI-1040 significantly inhibited NB cell proliferation in both MYCN -amplified and -non-amplified cell lines in a dose-dependent manner and has minimal effect on normal non-cancerous fibroblast cell lines (Figure 3A–C). The IC_50_ values determined are as follows SH-SY-5Y (9.7 μM), CHLA-255 (4.6 μM), SK-N-AS (3.8 μM), NGP (4.4 μM), LAN-5 (6.1 μM), CHLA-255-MYCN (4.5 μM).

Next, we used another proliferation assay and determined the effect of CI-1040 on NB colony formation and growth capacity, as colony formation ability is one of the most peculiar characteristics of solid tumor cancer cells. For this assay, we used two MYCN-amplified (NGP, IMR-32) and two MYCN non-amplified cell lines (SH-SY-5Y, CHLA-255) and treated them with CI-1040. Results demonstrated that CI-1040 significantly inhibited the colony formation and growth abilities of NB cells in a dose-dependent manner in comparison to controls (Figure 3D,E). Overall, these data indicate that CI-1040 significantly inhibits NB proliferation and colony formation in a dose-dependent manner.

### 3.3. CI-1040 Induces Apoptosis and Blocks Cell Cycle Progression in NB Cells

**Figure 4:**
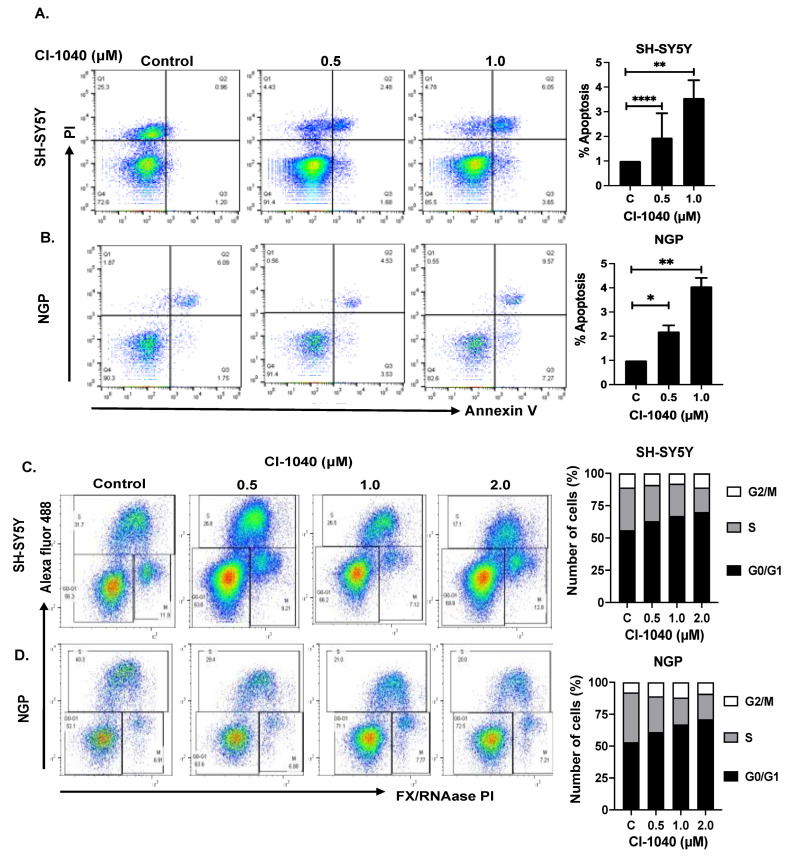
CI-1040 induces apoptosis and blocks cell cycle progression in NB cells. Apoptosis and cell cycle assays were performed using 2 NB cell lines including MYCN amplified (NGP) and MYCN non-amplified (SH-SY5Y) in response to CI-1040 treatment. (**A**,**B**) Representative flow cytometer images and the quantitative representation of the percentage of early apoptosis in (**A**) SH-SY5Y and (**B**) NGP cell lines. (**C**,**D**) Representative flow cytometer images and quantitative representation of the percentage of cells in each cell cycle phase. (**C**) SH-SY5Y and (**D**) NGP. * *p* < 0.05, ** *p* < 0.01, and **** *p* < 0.0001.

We further investigated the mechanism by which CI-1040 induces cytotoxicity and performed apoptosis and cell cycle assays using two NB cell lines, NGP and SH-SY5Y. Cells were treated with increasing concentrations of CI-1040, and the percentage of early apoptotic cells was measured, which was found to be significantly higher in CI-1040 treatment groups in comparison to controls. Our results demonstrate that 1 μM CI-1040 treatment induces apoptosis in SH-SY5Y cells to about 3.0-fold while in NGP to about 4.2-fold (Figure 4A,B), in contrast to the control treatments. Further, the cell cycle analysis showed that CI-1040 significantly inhibited the S phase in both NB cell lines to overall block NB cell cycle progression. Results demonstrated that 2 μM CI-1040 treatment inhibits cell cycle S phase by half in both NB cell lines SH-SY5Y and NGP (Figure 4C,D). The proportion of cells in the G0/G1 phase was increased while the cells in the S phase were decreased in CI-1040 treatment groups compared to control in a dose-dependent manner (Figure 4C,D). Overall, our data clearly demonstrate that CI-1040 induces apoptosis and blocks NB cell cycle progression at the DNA synthesis (S) phase in both MYCN-amplified and MYCN-non-amplified cell lines.

### 3.4. CI-1040 Inhibits NB Spheroid Tumor Growth

**Figure 5:**
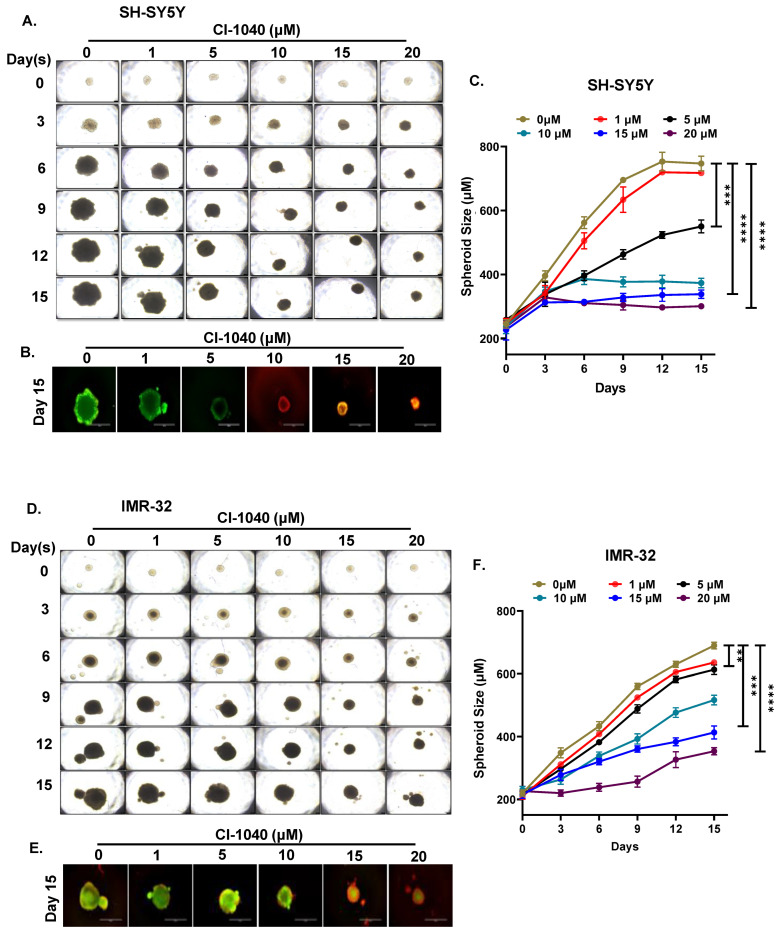
CI-1040 inhibits NB 3D spheroid tumor growth. NB 3D spheroids of MYCN amplified (IMR-32) and MYCN non-amplified (SH-SY5Y) cells were developed and treated with CI-1040 for 15 days. (**A**–**D**) Representative images of the 3D spheroid tumor growth at different days in response to CI-1040 treatment (**A**) SH-SY5Y (**D**) IMR-32. (**B**,**E**) Terminal day 3D spheroid tumor images stained with Calcein AM (Green; live cells) and EthD-III (Red; dead cells) fluorescence dyes. Size bar = 1 mm. (**B**) SH-SY5Y (E) IMR-32. (**C**,**F**) Quantitative 3D spheroid tumor growth showing reduction of tumor size in response to CI-1040 treatment (**C**) SH-SY5Y (**F**) IMR-32. ** *p* < 0.01, *** *p* < 0.001, and **** *p* < 0.0001.

**Figure 6:**
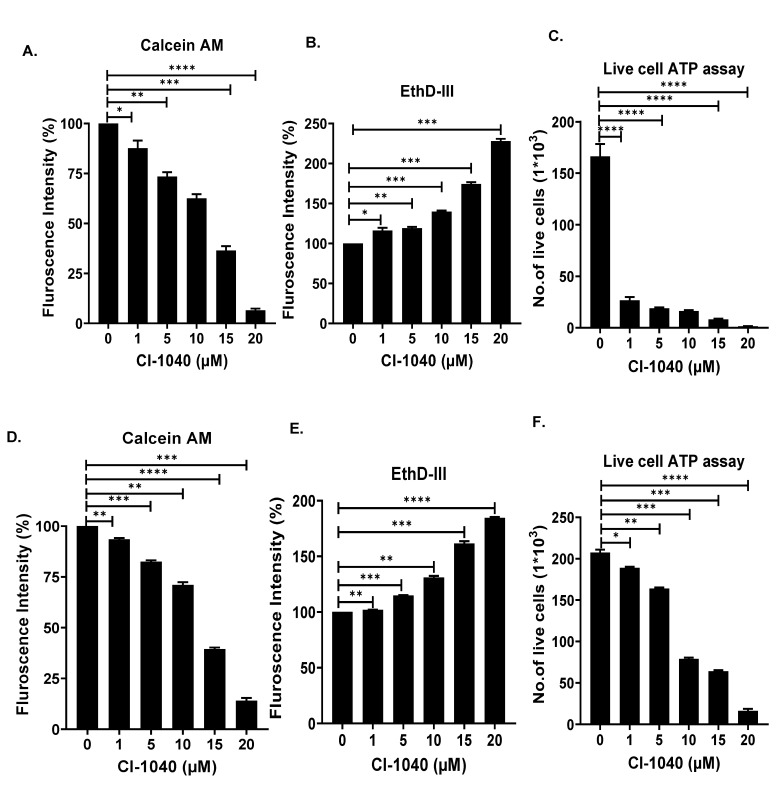
CI-1040 directly inhibits NB spheroidal live cells. NB 3D spheroid in Figure 5 were further analyzed for the effect of CI-1040 on tumor cell viability. (**A**,**D**) Quantitation of the percentage of live cells on final day (day 15) spheroids stained with Calcein AM (**A**) SH-SY5Y (**D**) IMR-32. (**B**,**E**) Quantitation of the percentage of dead cells stained with EthD-III. (**B**) SH-SY5Y (**E**) IMR-32. (**C**,**F**) Quantitation of the number of live cells using a live cell ATP release assay (**C**) SH-SY5Y (**F**) IMR-32. * *p* < 0.05, ** *p* < 0.01, *** *p* < 0.001, and **** *p* < 0.0001.

We further determined the effects of CI-1040 on the NB 3D spheroidal tumor models, which truly recapitulate the in vivo tumor growth patterns of solid tumor NB. Similar size spheroid tumors of SH-SY5Y, and IMR-32 cells were developed, randomized, and subjected to increasing doses of CI-1040. The size and growth of each spheroid were measured and imaged every third day up to 15 days (Figure 5A,D). Our spheroidal tumor data showed significant dose-dependent inhibition of tumor growth by CI-1040 in contrast to the control treatment (Figure 5C,F). Further, a significant dose-dependent reduction in the number of live cells and increase in dead cells were observed in terminal day fluorescence spheroid tumors stained with calcein-AM (green) and EthD-III (red), respectively, in both SH-SY5Y and IMR-32 (Figure 5B,E and Figure 6A,B,D,E). Further, these results were confirmed by quantifying the amount of ATP released from the live cells using a cell viability assay, which showed significant inhibition of the number of live tumor cells by CI-1040 (Figure 6C,F). Overall, our spheroidal tumor results demonstrate the efficacy and potency of CI-1040 in inhibiting NB tumor growth by directly inhibiting the live tumor cells.

### 3.5. CI-1040 Inhibits the MAPK Pathway in NB Cells

**Figure 7:**
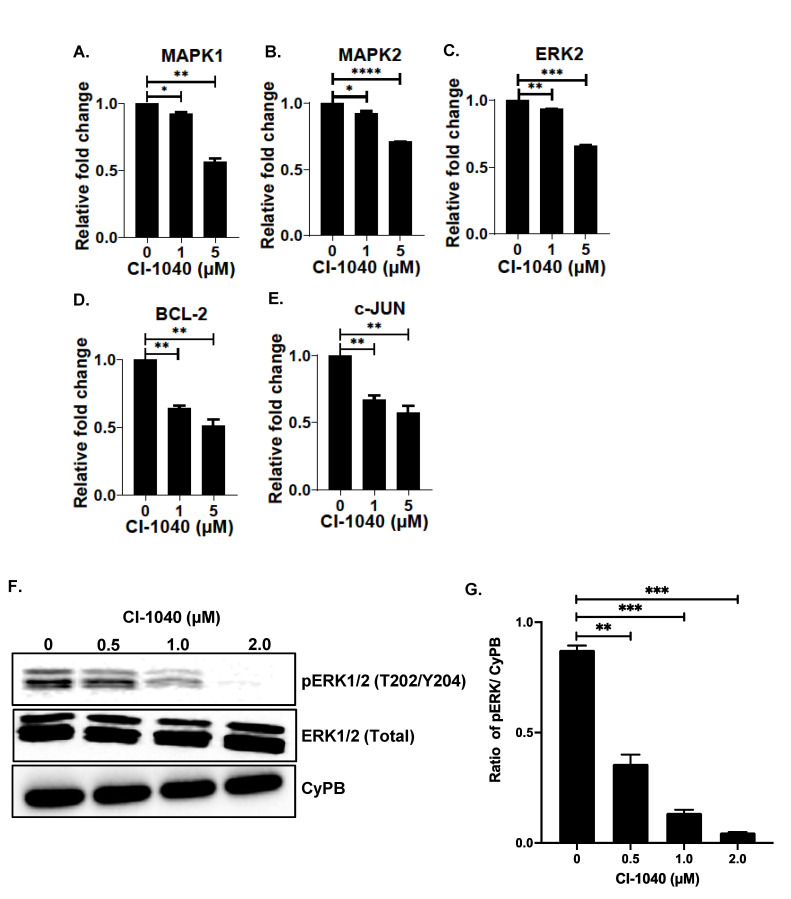
CI-1040 inhibits MEK/ERK pathway genes. (**A**–**E**) Gene expression analysis of different MAPK pathway and apoptosis related genes in response to CI-1040 treatment in SH-SY5Y cells. (**A**) *MAPK1.* (**B**) *MAPK2.* (**C**) *ERK2.* (**D**) *BCL-2.* (**E**) *c-JUN*. (**F**,**G**) Western blot analysis of total ERK1/2 and pERK1/2 (Thr202/Tyr204) in response to CI-1040 treatment. (**F**) Representative western blot images. (**G**) Densitometric analysis of pERK1/2 protein band as shown in F. * *p* < 0.05, ** *p* < 0.01, *** *p* < 0.001, and **** *p* < 0.0001.

To further determine the effect of CI-1040 on the MAPK pathway, we performed gene expression analysis on different MAPK and cell cycle-related genes. Our gene expression analysis revealed that CI-1040 significantly inhibits the mRNA expression of several MAPK pathway genes, such as *MAPK1*, *MAPK2*, and *ERK2*. CI-1040 also significantly inhibits gene expression of cell cycle-related genes such as *c-JUN* and *BCL-2* in a dose-dependent manner and in contrast to the controls (Figure 7A–E). Further, we performed Western blot analysis of the ERK1/2, as activation and phosphorylation of ERK1/2 at Thr202/Tyr204 is the final ultimate step of the MAPK pathway. Western blot analysis and densitometric quantitative analysis clearly demonstrated that CI-1040 significantly inhibits the phosphorylation of ERK1/2 at its Thr202/Tyr204 catalytic site in a dose-dependent manner and in contrast to the loading control CyPB (Figure 7F,G and Supplementary Figure S1). Additionally, CI-1040 showed no effect and reduction in the total ERK1/2, therefore further confirming the specificity as a MEK inhibitor by inhibiting the phosphorylation of ERK1/2. These data highlight the efficacy and potency of CI-1040 in inhibiting the Raf-MEK-ERK pathway by inhibiting the MEK-mediated ERK1/2 phosphorylation and activation.

## 4. Discussion

The MAPK pathway plays an essential signaling cascade that maintains cellular homeostasis under normal physiological conditions [15,32]. In addition, the MAPK pathway plays an important role in cell proliferation, cell growth, apoptosis, differentiation, angiogenesis, and tumor metastasis in multiple cancer types, including NB [33,34]. Previous studies have reported that ERK1/2 is crucial for cell survival and proliferation of NB, thyroid, breast, and prostate cancer stem cells [35,36,37,38]. Upregulation of MEK1/2 is mainly due to the catalytic activity or mutations of upstream regulators such as Ras, Raf, GRB2, and SOS [39,40]. MEK1/2 acts as a gatekeeper for the Ras-MEK-ERK pathway and transmits signals from multiple upstream regulators to the ERK1/2 [41].

In this study, we repurposed a highly specific small-molecule inhibitor CI-1040 in NB. CI-1040 is an ATP non-competitive MEK1/2 inhibitor that inhibits the phosphorylation and activation of the ERK1/2 [42,43]. CI-1040 is currently in phase II clinical trials (NCT00033384 and NCT00034827) for the treatment of advanced colorectal, lung, breast, and pancreatic cancer patients [28]. Our results demonstrated that CI-1040-mediated inhibition of ERK1/2 phosphorylation significantly inhibits NB growth. Previous research on CI-1040 established that this drug has an anti-proliferative activity against different types of cancer cells, including colon cancer and papillary thyroid carcinoma [25,44]. In the present study, we evaluated the anti-proliferative activity of MEK1/2 inhibitor CI-1040 on NB cells. CI-1040 inhibited NB cell growth and proliferation in a dose-dependent manner in both 2D cell culture and 3D tumor models.

Metabolic conversion of CI-1040 generates an anti-bacterial compound ATR-002, which interferes with the influenza life cycle by interrupting the Raf-MEK-ERK pathway in adenocarcinoma human alveolar basal epithelial cells [45]. We observed a dose-dependent induction of apoptosis and blockage of cell cycle progression at the S phase in NB cells by CI-1040 treatment. Similar results were observed in different cancer cells, including papillary thyroid carcinoma and multiple myeloma [25,46]. Further, our gene expression and Western blot analysis revealed that CI-1040 directly inhibits the expression of *MAPK*, *ERK*, *c-JUN*, and *BCL-2* genes and inhibits the phosphorylation of ERK1/2 protein. Similarly, CI-1040 has been shown to induce dexamethasone lethality in acute lymphoblastic leukemia cells through the pro-apoptotic molecule BCL-2 and MEK/ERK signaling pathway [47]. CI-1040 inhibits the expression of Cyclin D1 and phospho-p70S6K (Thr389) levels in the non-small-cell lung cancer (NSCLC) [48]. CI-1040, in combination with rapamycin and 17-AAG, has been shown to reduce prostate cancer metastasis [26]. Similar to our results, three-dimensional (3D) spheroidal tumor studies in breast cancer revealed that the cytostatic effect of CI-1040 is high in 3D models compared to 2D models, and CI-1040 specifically inhibits the MEK/ERK pathway [27]. We also observed a differential specificity of CI-1040 in inhibiting the NB 2D and 3D tumor models, with higher specificity for 3D spheroid tumors.

## 5. Conclusions

In conclusion, the Ras-MEK-ERK pathway is one of the most important cell signaling pathways that dysregulate in many cancers, including NB. Our results clearly demonstrate the potency of CI-1040 in inhibiting NB proliferation and growth in NB cells and spheroidal tumors. We demonstrated that CI-1040, in a dose-dependent manner, induces apoptosis and arrests cell cycle progression in NB cells, inhibits the transcription of multiple oncogenes, and inhibits the phosphorylation and activation of ERK1/2 in NB. Overall, our results demonstrated that targeting the Ras-MEK-ERK pathway by CI-1040 is an effective therapeutic approach for NB and for other MEK/ERK pathway-driven cancers.

## Figures and Tables

**Table 1:** RT-qPCR primers used in the study.

Gene	Forward Primer (5′-3′)	Reverse Primer (5′-3′)
*MAPK1*	TACACCAACCTCTCGTACATCG	CATGTCTGAAGCGCAGTAAGATT
*MAPK2*	GGCAGCTACCTCAGGAATGAC	CCAGTGGCATGGTAAATCTCC
*ERK2*	ACGGCATGGTTTGCTCTGCTTATG	TCATTTGCTCAATGGTTGGTGCCC
*c-Myc*	TACACTAACATCCCACGCTCTG	CGCATCCTTGTCCTGTGAGT
*c-JUN*	CCCCAAGATCCTGAAACAGA	CCGTTGCTGGACTGGATTAT
*BCL-2*	GTGGATGACTGAGTACCTGAAC	GAGACAGCCAGGAGAAATCAA
*GAPDH*	CACCATCTTCCAGGAGCGAG	TGATGACCCTTTTGGCTCCC

## Data Availability

The data presented in this study are available in this article and in the supplementary material. The patient dataset was re-analyzed by using the R2 database (https://hgserver1.amc.nl (accessed on 16 July 2022)).

## Supplementary Materials

The following supporting information can be downloaded at: https://www.mdpi.com/article/10.3390/curroncol29090512/s1, Figure S1: Full Western blot images.

## References

[1] Eleveld T.F., Oldridge D.A., Bernard V., Koster J., Colmet Daage L., Diskin S.J., Schild L., Bentahar N.B., Bellini A., Chicard M. (2015). Relapsed neuroblastomas show frequent RAS-MAPK pathway mutations. Nat. Genet..

[2] Ritenour L.E., Randall M.P., Bosse K.R., Diskin S.J. (2018). Genetic susceptibility to neuroblastoma: Current knowledge and future directions. Cell Tissue Res..

[3] Anderson J., Majzner R.G., Sondel P.M. (2022). Immunotherapy of Neuroblastoma: Facts and Hopes. Clin. Cancer Res..

[4] Kholodenko I.V., Kalinovsky D.V., Doronin I.I., Deyev S.M., Kholodenko R.V. (2018). Neuroblastoma Origin and Therapeutic Targets for Immunotherapy. J. Immunol. Res..

[5] Heinly B.E., Grant C.N. (2022). Cell Adhesion Molecules in Neuroblastoma: Complex Roles, Therapeutic Potential. Front. Oncol..

[6] Zafar A., Wang W., Liu G., Wang X., Xian W., McKeon F., Foster J., Zhou J., Zhang R. (2021). Molecular targeting therapies for neuroblastoma: Progress and challenges. Med. Res. Rev..

[7] Han J., Liu Y., Yang S., Wu X., Li H., Wang Q. (2021). MEK inhibitors for the treatment of non-small cell lung cancer. J. Hematol. Oncol..

[8] Franklin D.A., Sharick J.T., Ericsson-Gonzalez P.I., Sanchez V., Dean P.T., Opalenik S.R., Cairo S., Judde J.G., Lewis M.T., Chang J.C. (2020). MEK activation modulates glycolysis and supports suppressive myeloid cells in TNBC. JCI Insight.

[9] Bu R., Siraj A.K., Masoodi T., Parvathareddy S.K., Iqbal K., Al-Rasheed M., Haqawi W., Diaz M., Victoria I.G., Aldughaither S.M. (2021). Recurrent Somatic MAP2K1 Mutations in Papillary Thyroid Cancer and Colorectal Cancer. Front. Oncol..

[10] Greengard E.G. (2018). Molecularly Targeted Therapy for Neuroblastoma. Children.

[11] Braicu C., Buse M., Busuioc C., Drula R., Gulei D., Raduly L., Rusu A., Irimie A., Atanasov A.G., Slaby O. (2019). A Comprehensive Review on MAPK: A Promising Therapeutic Target in Cancer. Cancers.

[12] Moon H., Ro S.W. (2021). MAPK/ERK Signaling Pathway in Hepatocellular Carcinoma. Cancers.

[13] Guo Y.J., Pan W.W., Liu S.B., Shen Z.F., Xu Y., Hu L.L. (2020). ERK/MAPK signalling pathway and tumorigenesis. Exp. Ther. Med..

[14] Muta Y., Matsuda M., Imajo M. (2019). Divergent Dynamics and Functions of ERK MAP Kinase Signaling in Development, Homeostasis and Cancer: Lessons from Fluorescent Bioimaging. Cancers.

[15] Eblen S.T. (2018). Extracellular-Regulated Kinases: Signaling From Ras to ERK Substrates to Control Biological Outcomes. Adv. Cancer Res..

[16] Eleveld T.F., Schild L., Koster J., Zwijnenburg D.A., Alles L.K., Ebus M.E., Volckmann R., Tijtgat G.A., van Sluis P., Versteeg R. (2018). RAS-MAPK Pathway-Driven Tumor Progression Is Associated with Loss of CIC and Other Genomic Aberrations in Neuroblastoma. Cancer Res..

[17] Woodfield S.E., Zhang L., Scorsone K.A., Liu Y., Zage P.E. (2016). Binimetinib inhibits MEK and is effective against neuroblastoma tumor cells with low NF1 expression. BMC Cancer.

[18] Subramonian D., Phanhthilath N., Rinehardt H., Flynn S., Huo Y., Zhang J., Messer K., Mo Q., Huang S., Lesperance J. (2020). Regorafenib is effective against neuroblastoma in vitro and in vivo and inhibits the RAS/MAPK, PI3K/Akt/mTOR and Fos/Jun pathways. Br. J. Cancer.

[19] Sugiura R., Satoh R., Takasaki T. (2021). ERK: A Double-Edged Sword in Cancer. ERK-Dependent Apoptosis as a Potential Therapeutic Strategy for Cancer. Cells.

[20] Dorel M., Klinger B., Mari T., Toedling J., Blanc E., Messerschmidt C., Nadler-Holly M., Ziehm M., Sieber A., Hertwig F. (2021). Neuroblastoma signalling models unveil combination therapies targeting feedback-mediated resistance. PLoS Comput. Biol..

[21] Takeuchi Y., Tanaka T., Higashi M., Fumino S., Iehara T., Hosoi H., Sakai T., Tajiri T. (2018). In vivo effects of short- and long-term MAPK pathway inhibition against neuroblastoma. J. Pediatr. Surg..

[22] Cheng Y., Tian H. (2017). Current Development Status of MEK Inhibitors. Molecules.

[23] Liu F., Yang X., Geng M., Huang M. (2018). Targeting ERK, an Achilles’ Heel of the MAPK pathway, in cancer therapy. Acta Pharm. Sin. B.

[24] Allen L.F., Sebolt-Leopold J., Meyer M.B. (2003). CI-1040 (PD184352), a targeted signal transduction inhibitor of MEK (MAPKK). Semin. Oncol..

[25] Henderson Y.C., Ahn S.H., Clayman G.L. (2009). Inhibition of the growth of papillary thyroid carcinoma cells by CI-1040. Arch. Otolaryngol. Head Neck Surg..

[26] Ding G., Feng C., Jiang H., Ding Q., Zhang L., Na R., Xu H., Liu J. (2013). Combination of rapamycin, CI-1040, and 17-AAG inhibits metastatic capacity of prostate cancer via Slug inhibition. PLoS ONE.

[27] Li Q., Chow A.B., Mattingly R.R. (2010). Three-dimensional overlay culture models of human breast cancer reveal a critical sensitivity to mitogen-activated protein kinase kinase inhibitors. J. Pharmacol. Exp. Ther..

[28] Rinehart J., Adjei A.A., Lorusso P.M., Waterhouse D., Hecht J.R., Natale R.B., Hamid O., Varterasian M., Asbury P., Kaldjian E.P. (2004). Multicenter phase II study of the oral MEK inhibitor, CI-1040, in patients with advanced non-small-cell lung, breast, colon, and pancreatic cancer. J. Clin. Oncol..

[29] Lorusso P.M., Adjei A.A., Varterasian M., Gadgeel S., Reid J., Mitchell D.Y., Hanson L., DeLuca P., Bruzek L., Piens J. (2005). Phase I and pharmacodynamic study of the oral MEK inhibitor CI-1040 in patients with advanced malignancies. J. Clin. Oncol..

[30] Chilamakuri R., Rouse D.C., Yu Y., Kabir A.S., Muth A., Yang J., Lipton J.M., Agarwal S. (2022). BX-795 inhibits neuroblastoma growth and enhances sensitivity towards chemotherapy. Transl. Oncol..

[31] Chilamakuri R., Agarwal S. (2022). Dual Targeting of PI3K and HDAC by CUDC-907 Inhibits Pediatric Neuroblastoma Growth. Cancers.

[32] Keshet Y., Seger R. (2010). The MAP kinase signaling cascades: A system of hundreds of components regulates a diverse array of physiological functions. Methods Mol. Biol..

[33] Healy J.R., Hart L.S., Shazad A.L., Gagliardi M.E., Tsang M., Elias J., Ruden J., Farrel A., Rokita J.L., Li Y. (2020). Limited antitumor activity of combined BET and MEK inhibition in neuroblastoma. Pediatr. Blood Cancer.

[34] Jin X.F., Spottl G., Maurer J., Nolting S., Auernhammer C.J. (2021). Antitumoral Activity of the MEK Inhibitor Trametinib (TMT212) Alone and in Combination with the CDK4/6 Inhibitor Ribociclib (LEE011) in Neuroendocrine Tumor Cells In Vitro. Cancers.

[35] Paramanantham A., Jung E.J., Go S.I., Jeong B.K., Jung J.M., Hong S.C., Kim G.S., Lee W.S. (2021). Activated ERK Signaling Is One of the Major Hub Signals Related to the Acquisition of Radiotherapy-Resistant MDA-MB-231 Breast Cancer Cells. Int. J. Mol. Sci..

[36] Okuda K.S., Keyser M.S., Gurevich D.B., Sturtzel C., Mason E.A., Paterson S., Chen H., Scott M., Condon N.D., Martin P. (2021). Live-imaging of endothelial Erk activity reveals dynamic and sequential signaling events during regenerative angiogenesis. Elife.

[37] Leng Y., Chen Z., Ding H., Zhao X., Qin L., Pan Y. (2021). Overexpression of microRNA-29b inhibits epithelial-mesenchymal transition and angiogenesis of colorectal cancer through the ETV4/ERK/EGFR axis. Cancer Cell Int..

[38] Zhong L., Li Y., Xiong L., Wang W., Wu M., Yuan T., Yang W., Tian C., Miao Z., Wang T. (2021). Small molecules in targeted cancer therapy: Advances, challenges, and future perspectives. Signal Transduct. Target. Ther..

[39] Lake D., Correa S.A., Muller J. (2016). Negative feedback regulation of the ERK1/2 MAPK pathway. Cell Mol. Life Sci..

[40] Pudewell S., Wittich C., Kazemein Jasemi N.S., Bazgir F., Ahmadian M.R. (2021). Accessory proteins of the RAS-MAPK pathway: Moving from the side line to the front line. Commun. Biol..

[41] Caunt C.J., Sale M.J., Smith P.D., Cook S.J. (2015). MEK1 and MEK2 inhibitors and cancer therapy: The long and winding road. Nat. Rev. Cancer.

[42] Wu P.K., Park J.I. (2015). MEK1/2 Inhibitors: Molecular Activity and Resistance Mechanisms. Semin. Oncol..

[43] Barrett S.D., Bridges A.J., Dudley D.T., Saltiel A.R., Fergus J.H., Flamme C.M., Delaney A.M., Kaufman M., LePage S., Leopold W.R. (2008). The discovery of the benzhydroxamate MEK inhibitors CI-1040 and PD 0325901. Bioorg. Med. Chem. Lett..

[44] Liu D., Liu Z., Jiang D., Dackiw A.P., Xing M. (2007). Inhibitory effects of the mitogen-activated protein kinase kinase inhibitor CI-1040 on the proliferation and tumor growth of thyroid cancer cells with BRAF or RAS mutations. J. Clin. Endocrinol. Metab..

[45] Bruchhagen C., Jarick M., Mewis C., Hertlein T., Niemann S., Ohlsen K., Peters G., Planz O., Ludwig S., Ehrhardt C. (2018). Metabolic conversion of CI-1040 turns a cellular MEK-inhibitor into an antibacterial compound. Sci. Rep..

[46] VanBrocklin M.W., Verhaegen M., Soengas M.S., Holmen S.L. (2009). Mitogen-activated protein kinase inhibition induces translocation of Bmf to promote apoptosis in melanoma. Cancer Res..

[47] Rambal A.A., Panaguiton Z.L., Kramer L., Grant S., Harada H. (2009). MEK inhibitors potentiate dexamethasone lethality in acute lymphoblastic leukemia cells through the pro-apoptotic molecule BIM. Leukemia.

[48] Mahoney C.L., Choudhury B., Davies H., Edkins S., Greenman C., Haaften G., Mironenko T., Santarius T., Stevens C., Stratton M.R. (2009). LKB1/KRAS mutant lung cancers constitute a genetic subset of NSCLC with increased sensitivity to MAPK and mTOR signalling inhibition. Br. J. Cancer.

