# Supplementary figures and images for: Direct Targeting of the Raf-MEK-ERK Signaling Cascade Inhibits Neuroblastoma Growth
Source: Curr Oncol. 2022 Sep 10;29(9):6508–22. doi: 10.3390/curroncol29090512 (PMC9497977; doi:10.3390/curroncol29090512)

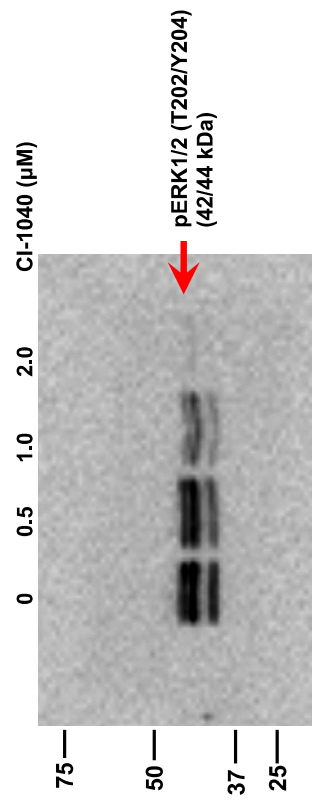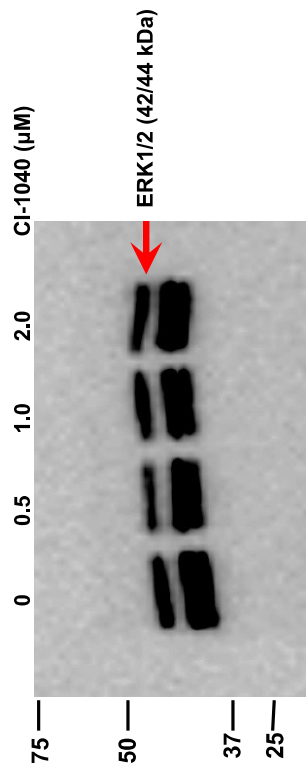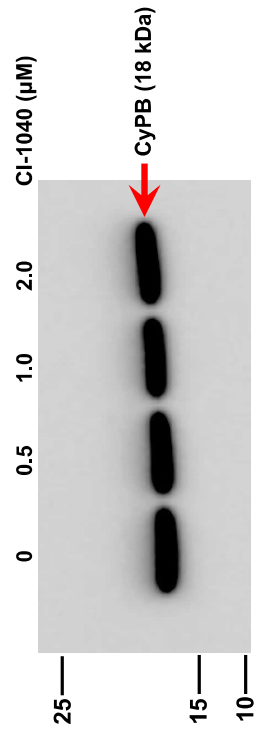

Supplement: Supplementary file 1 [file curroncol-29-00512-s001.zip › curroncol-1892703-supplementary.pdf]
